# Patho-epidemiological study on Genotype-XIII Newcastle disease virus infection in commercial vaccinated layer farms

**DOI:** 10.14202/vetworld.2015.372-381

**Published:** 2015-03-21

**Authors:** J. H. Khorajiya, Sunanda Pandey, Priya D. Ghodasara, B. P. Joshi, K. S. Prajapati, D. J. Ghodasara, R. A. Mathakiya

**Affiliations:** 1 Department of Veterinary Pathology, College of Veterinary Science and Animal Husbandry, Anand Agricultural University, Anand, Gujarat - 388 001, India; 2 Department of Veterinary Microbiology, College of Veterinary Science and Animal Husbandry, Anand Agricultural University, Anand, Gujarat - 388 001, India

**Keywords:** genotype, histopathology, intra-cerebral pathogenicity index, Newcastle disease

## Abstract

**Aim:**

The present research work was carried out to study the patho-epidemiological aspects of Genotype-XIII Newcastle disease virus (NDV) infection in commercial layer in and around Anand, Gujarat. As the outbreaks have reported in vaccinated flocks, it was felt necessary to study the disease with respect to its changing pathogenicity and relevant aspects.

**Materials and Methods:**

The study comprised of patho-epidemiology of Newcastle disease (ND) by information collected from different layer farms suffering from the disease in relation to incidence pattern and mortality, duration of mortality, susceptible age, and loss due to production performance. Clinical signs were recorded based on observations. During post-mortem, gross lesions were also recorded. For histopathological examination visceral organs according to lesions were collected in 10% formalin and processed slide stained by hematoxylin and eosin for microscopic examination. Cultivation of virus was done in embryonated specific pathogen-free (SPF) eggs of 9-11 days and isolation of virus was done for haemagglutination (HA) and haemagglutination inhibition (HI) test and to identify pathotype of virus by intracerebral pathogenicity index (ICPI) test to determine the virulence of virus. The Genotype-XIII NDV was confirmed by F gene sequence and whole genome sequence.

**Results:**

During the study mortality due to ND was recorded in 13 layer flocks in spite of routine vaccination, which usually contain Genotype-II strain of virus. The mortality was observed as high as above 50% with an average of 21.21%. The susceptible age for disease was found to be 6-14 weeks. The duration of mortality observed was 23 days. The disease resulted in a significant reduction in body weight, feed intake and drop in egg production. Majority of the outbreaks appeared during extremely hot months of April to June. Greenish diarrhoea was frequently seen in birds that survived early in infection. Mortality continued for 2-3 weeks and reduced with appearance of torticollis. Gross lesions were characterized by multifocal to diffuse hemorrhages around proventricular glands, necrotic (diphtheritic) haemorrhagic ulcers throughout the intestine, disseminated multiple foci of necrosis and pin-point hemorrhages in the spleen parenchyma. The microscopic lesions include focal to diffuse hemorrhages, diffuse infiltration of mononuclear cells, necrosis, and degeneration in visceral organs. All the 13 farm samples (n=13) resulted in death of all the embryos following incubation up to 72 h post-inoculation. All the 13 allantois fluids from field samples along with F and R2B vaccine sample were found positive for HA activity, which was further confirmed by HI using known NDV serum. The values of ICPI were 2.0 which were indicative of velogenic nature of the field NDV strain.

**Conclusion:**

The study indicated that presently available live and attenuated vaccines which include Genotype-II NDV have failed in protecting the flocks against Genotype-XIII and resulted in outbreaks with mortality above 50%. ICPI score of 2.0 confirmed that the present outbreaks were due to Genotype-XIII NDV, which is velogenic in nature.

## Introduction

Indian poultry industry is one of the fastest growing segments of the agricultural sector in India. Presently India has emerged as the World’s second largest poultry market with an annual growth of more than 14%, producing 66 billion eggs in 2012 [1]. However, a marked increase in poultry population along with the drastic changes in the husbandry practices resulted in an increase in the prevalence and altered patterns of the poultry diseases. The presence of diseases in the poultry flock has reflected in inferior performance and has consistently been a major limiting factor to profitable production. Newcastle disease (ND) is an Office International des epizootics (OIE) listed infection and considered as one of the most important diseases of chicken [2]. It causes huge economic losses to farmers in the form of mortality and heavy production loss. Depending upon the pathotype and susceptibility of birds the mortality varies from 0% to 100% [3]. Since its first report in India between 1928 and 1930 at Ranikhet [4] and Madras-Chennai [5] it still remains endemic in India and outbreaks are reported regularly in spite of vaccination. The OIE defines ND as an infection caused by a highly virulent Avian Paramyxo virus-1 (APMV-1), an isolate that has either an intra-cerebral pathogenicity index (ICPI) of at least 0.7 in day-old chicks, or an amino acid sequence with multiple basic amino acids (at least three arginine (R) or lysine (K) residues at the C-terminus of the F2 protein starting at position 113, along with a phenylalanine at position 117 at N-terminus of the F1 protein. APMV-1 is a member of the genus Avulavirus in the family Paramyxoviridae of the order Mononegavirales [6]. The Paramyxoviruses isolated from avian species have been classified into eleven subtypes designated APMV-1 to APMV-11 by serological testing and phylogenetic analysis [7]. Based on phylogenetic analysis with the partial hypervariable nucleotide sequences of the F gene, NDV strains have been classified into 18 genotypes (Class II, Genotypes I-XVIII). Recent reports from West and Central Africa described the presence of novel VNDV strains belonging to new genetic lineages closely related to Genotype VII namely XIV, XVII and XVIII [8].

ND is endemic in many parts of the world including countries in Asia, the Middle East, Africa, and Central and South America. In terms of economic impact, no other poultry virus comes even close to NDV. It may represent a bigger drain on the livestock economy than any other animal virus. In spite of a stringent vaccination policy against ND, outbreaks still occur in many countries.

During recent years outbreaks of ND in commercial layer farms were observed around Anand, Gujarat. Failure of vaccination programme also suspected emergence of the new genotype of NDV. As the outbreaks have reported in vaccinated flocks, it was felt necessary to study the disease with respect to its changing pathogenicity and related relevant aspects.

## Materials and Methods

### Ethical approval

The present study was approved by Institutional Animal Ethics Committee. The authors have taken permission from each poultry farm owner to publish data.

### Epidemiological study

The information regarding epidemiological study was obtained by making personal visits to the commercial layer farms situated around Anand, Gujarat where mortality was reported due to ND with regular vaccination programme. The information like name and location of farm, strength of flock, age of the flock when ND was first detected, mortality pattern, duration of mortality (Period for which mortality continues in the farm during outbreak), effect on body weight, production drop, effect on feed consumption, history of vaccination, type of vaccination were recorded. Total 13 layer farms were visited where mortality due to Newcastle disease virus (NDV) was reported.

### Gross pathology

During the outbreak of disease in the layer farms, the ailing birds were examined for clinical signs if any and detailed post-mortem was carried out from the carcass in the Department of Pathology and gross pathological lesions were recorded. The gross pathological lesions were recorded and characterized depending upon the severity of the disease as well as concominent other lesions suggestive of secondary bacterial infections like *Escherichia coli* or Mycoplasma.

### Histopathological study

For histopathological examination, tissues from visceral organs like lung, trachea, liver, spleen, kidney, proventriculus, intestine, caecal tonsils, bursa of Fabricious and brain were collected in 10% neutral buffered formalin and processed by paraffin embedding technique. Sections were cut at 5-6 µ thickness with automatic section cutting machine (Leica, Germany) and stained with haematoxylin and eosin (H and E) [9]. The H and E stained sections were observed under the light microscope, and lesions were recorded.

### Collection of samples for virological study (NDV propagation in egg embryo)

At the time of post-mortem examination, the carcasses showing gross lesions suggestive of ND were selected for collection of samples for detection of NDV i.e. Propagation and cultivation of NDV in specific pathogen-free egg embryo. After taking necessary aseptic precautions, the pooled tissue samples of trachea, lung, spleen, proventriculus, caecal tonsils and intestine representative of each layer farms were collected separately in sterile petridish for further processing. A total of 13 samples from 13 farms were collected, and the tissues from each farm were pooled and processed as a single sample.

### NDV

Reference virus strains (F and R2B) of NDV obtained from the Vaccine Institute, Gandhinagar were used for control study.

### Cultivation of NDV in egg embryo

Inoculation in embryo through allantoic route and collection of allantoic fluid carried out as per the method described in OIE Terrestrial Manual 2012 [10].

### HA and HI tests

To isolate virus both the test was carried out as per the method described in OIE Terrestrial Manual 2012.

### Pathogenicity index

ICPI is an accepted *in vivo* test for the assessment of virulence of NDV in 1-day-old chicks because of its established accuracy and sensitivity (OIE Terrestrial Manual 2012). During the study, representative samples of different layer farms were inoculated in day old chicks and were pathotyped performing ICPI to evaluate the virulence of the field isolates as stated by Alexander and Senne [11].

The present field isolates obtained during field outbreaks of ND from layer and broiler farms were submitted to Department of Microbiology for F gene sequence and to Department of Animal Biotechnology for whole genome sequencing. The Genome length found was 15192nt. The phylogenetic analysis and evolutionary distances placed these isolates in Genotype XIII (XIIIb) with the available latest sequences in the gene bank.

## Results

The results obtained from this study have been summarized under the following headings and are described with the help of different tables.

### Epidemiological studies

ND was reported in thirteen layer farms around Anand, Gujarat, India in spite of routine vaccination programme during the period March 2013 to April 2014. Anand is located at 22.57°N 72.93°E. The day temperature in winter is around 83°F and at night is 53°F. Summers are extremely hot, with the day temperature being 115°F, and the night temperature being 90°F. In Gujarat, monsoon is generally hot and utterly humid. The temperature at day is 100°F but at night it falls down to 80°F.

### Strength and age of the flock when ND first detected

The strength of the affected flocks in different farms ranged between 4320 and 33764 birds (Table-1). The outbreaks of ND in all the flocks studied were in the age group ranging between 5 and 36 weeks. The disease was noticed as early as 33 days to as later on 252 days of age. All the affected flocks were housed in cage system of management.

**Table-1:** Information regarding flock strength, age of the flock when ND first detected, total mortality, duration of mortality and vaccination history in commercial layer farms affected with ND.

Name and location of farm	Flock strength	Age of the flock when ND first detected (days)	Total no of bird died	Mortality %	No of days for which mortality continues during outbreak (days)	Vaccination history		

						Type of vaccine	Schedule of vaccination (weeks)	Dose and route
Arpan Poultry Farm, Navli	8150	33	2518	25	24	Lasota	1	Drinking water
						ND Killed	1	0.3 ml S/C
Bhoomi Poultry Farm, Sarsa	33764	34	18194	53.88	46	Lasota	1	Drinking water
						ND killed	1	0.3 ml S/C
Narayan Poultry Farm, Navli	6361	34	844	13.26	26	Lasota	1	Drinking water
						ND Killed	1	0.25 ml S/C
Samarpan Poultry Farm, Navli	15319	36	3912	25.53	20	Lasota	1	Drinking water
						ND killed	1	0.25 ml S/C
						Lasota	5	Drinking water
Radhekrishna Poultry Farm, Verakhadi	15240	43	5880	38.58	23	Lasota	1	Drinking water
						ND killed	1	0.25 ml S/C
						Lasota	5	Eye Drop
J.K Poultry Farm, Sarsa	11229	60	2927	26.06	27	Lasota	1	Drinking water
						ND killed	1	0.25 ml S/C
						Lasota	5	Drinking water
Shradha Poultry Farm Flock -B, Sarsa	5283	60	1015	19.21	22	Lasota	1	Drinking water
						ND killed	1	0.25 ml S/C
						Lasota	5	Drinking water
Yogi Poultry Farm, Sarsa	4320	68	395	9.14	13	Lasota	1	Drinking water
						ND Killed	1	0.25 ml S/C
						Lasota	5	Drinking water
Honest Poultry Farm Flock No- B, Sarsa	11597	74	1640	14.14	25	Lasota	1	Drinking water
						ND killed	1	0.25 ml S/C
						Lasota	5	Drinking water
						R2B	10	0.5 m. I/M
Shradha Poultry Farm Flock No-A, Sarsa	5335	88	2927	16.1	25	Lasota	1	Drinking water
						ND killed	1	0.25 ml S/C
						Lasota	5	Drinking water
						R2B	10	0.5 m. I/M
Adarsh Poultry Farm, Kasor	13911	97	2594	18.64	15	Lasota	1	Drinking water
						ND killed	1	0.25 ml S/C
						Lasota	5	Drinking water
						R2B	10	0.5 m. I/M
Honest Poultry Farm Flock No-A, Sarsa	10512	244	686	6.53	17	Lasota	1	Drinking water
						ND killed	1	0.25 ml S/C
						Lasota	5	Drinking water
						R2B	10	0.5 m. I/M
						Lasota	20	Drinking water
						ND killed	20	0.5 ml S/C
						Lasota	35	Drinking water
Keval Poultry Farm, Sarsa	12087	252	1166	9.65	21	Lasota	1	Drinking water
						ND killed	1	0.25 ml S/C
						Lasota	5	Drinking water
						R2B	10	0.5 m. I/M
						Lasota	20	Drinking water
						ND killed	20	0.5 ml S/C
						Lasota	35	Drinking water

ND=Newcastle disease

### Mortality

Mortality due to ND in all the thirteen flocks was ranged between 6.53 and 53.88%. The overall mortality was 21.21%. Among different layer birds mortality in layer chicks ranged from 13.26% to 53.88%, in growers 9.14-19.21%, and in layers 6.53-9.65% (Table-1). Younger flocks had higher mortality due to low vaccinal immunity resulting from less number of vaccinations. The death was reported daily without any control in spite of vaccination and medication. The duration of mortality recorded in all the layer farms was as short duration as 13 days at Yogi poultry Farm, Sarsa and as long duration as 46 days at Bhoomi poultry Farm, Sarsa. The overall duration of mortality was 23 days (Table-1).

### Vaccination history

As per available records on each of the affected farms it was noticed that the affected layer flocks were vaccinated with Lasota (D/W) and lentogenic ND Killed 0.25 ml (S/C) at neck region in 1st week (Flock no. 1, 2, 3) further Lasota boostering was done at 5^th^ week (Flock no. 4, 5, 6, 7 and 8), R_2_B 0.5 ml (I/M) at 10th week (Flock No. 9, 10 and 11), Lasota (D/W) and Lentogenic ND killed 0.5 ml (S/C) during prelay stage at 20^th^ week and Lasota (D/W) at 35^th^ weeks of age (Flock No. 12 and 13). The vaccination schedule was followed as per standard guidelines using cold chain.

### Effect on feed consumption

The entire affected layer flocks revealed reduction in feed intake ranging from 3.46% to 46.91%. In the majority of the farms (10/13) feed intake was reduced above 17%, whereas in three farms it was reduced below 10% in comparison to the standard feed intake (Table-2).

**Table-2:** Information regarding Name of the farm, effect on body weight, effect on feed consumption and seasonal distribution in commercial layer farms affected with ND.

Name and location of farm	Average feed consumption during course of disease (g)	Standard normal feed intake BV=300 (g)	Percent Reduction in feed intake during course of disease	Average body weight during course of disease (g)	Standard normal body weight in gram BV=300	Percent reduction in body weight during course of disease	Month of outbreak
Arpan Poultry Farm, Navli	38.99	42.56	8.38	398	462	13.85	February
Bhoomi Poultry Farm, Sarsa	29.54	46.93	37.05	498	626	20.44	June
Narayan Poultry Farm, Navli	29.59	43.88	32.56	365	506	27.86	May
Samarpan Poultry Farm, Navli	39.36	43.57	10	335	480	30.20	January
Radhekrishna Poultry Farm, Veraikhadi	32.64	47.26	30.93	346	590	42.37	May
J.K Poultry Farm, Sarsa	49.87	51.66	3.46	636	813	21.77	May
Shradha Poultry Farm Flock -B, Sarsa	40.22	50.86	20.92	632	786	19.59	June
Yogi Poultry Farm, Sarsa	35.24	51.46	31.51	640	822	22.14	May
Honest Poultry Farm Flock No-B, Sarsa	29.39	55.36	46.91	761	954	20.48	May
Shradha Poultry Farm Flock No-A, Sarsa	49.24	59.44	17.16	876	1012	13.43	April
Adarsh Poultry Farm, Kasor	49.94	60.82	17.88	900	1107	18.69	June
Honest Poultry Farm Flock No- A, Sarsa	68.92	96.02	28.23	1220	1220	0.0	April
Keval Poultry Farm, Sarsa	78.71	95.71	17.76	1220	1220	0.0	April

ND=Newcastle disease

### Effect on body weight

The entire affected layer flocks revealed depression in body weight ranging from 13.85% to 42.37%. Majority of farms (11/13) revealed depression in body weight above 19% during the course of the disease as compared to standard body weight as per the guidelines given by hatchery (Table-2).

### Seasonal distribution

During the study period, it was observed that majority of the outbreaks (11/13) were reported in the summer months of April to June while only two farms showed the presence of disease in the month of January and February. The seasonal distribution pattern indicated that birds were more prone for the disease in summer months than winter months (Table-2).

### Effect on production

Out of total thirteen flocks affected with the disease, only two farms were in laying phase. As per the available data of production performance, both these flocks revealed drop in egg production during the course of the disease. The drop in egg production was from 89.28% to 56.44% in Keval Poultry farm Sarsa, and from 89.4% to 68.0% in Honest Poultry Farm, Flock No-A, Sarsa. Production reached up to 84% after 6 weeks at Keval Poultry farm Sarsa, and up to 86% after 4 weeks at Honest Poultry Farm, Flock No-A, Sarsa. But none of the farms reached up to original production more than 86% during laying phase. Both these laying flocks showed 20-30% drop in hen day egg production. There was a slow recovery after a month but failed to reach original production level. The appearance of lethargy eggs and eggshell breakage which are usually seen with classical ND outbreaks were not severe in the present outbreaks.

### Clinical signs

**Figure-1:**
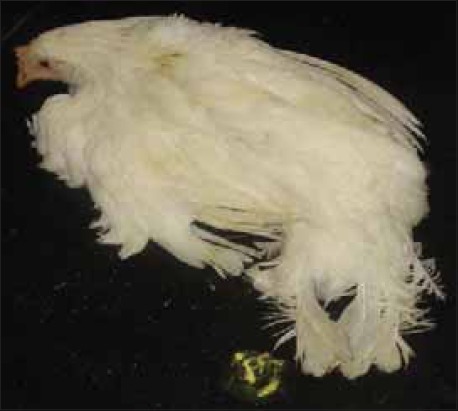
Layer bird affected with Newcastle disease showing greenish diarrhoea and depression.

**Figure-2:**
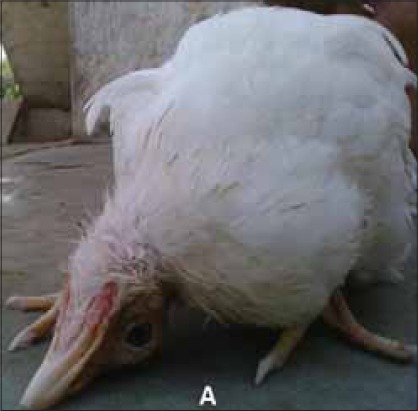
Layer bird affected with Newcastle disease showing characteristic symptoms of torticollis (twisted neck and paralysis).

At the beginning, the disease appeared suddenly with high mortality without any clinical signs in the entire affected layer. Subsequently clinical signs appeared gradually. They include reduced feed intake, listlessness, increased respiration, Greenish diarrhoea with soiled feathers of vent, dehydration, loss in body weight, conjunctivitis, prostration and increasing mortality. Some of the flocks also showed oedema around eyes and head. Greenish diarrhoea (Figure-1) was frequently seen in birds that survived early in infection. Mortality continued for 2-3 weeks and reduced with appearance of torticollis. Birds with torticollis (Figure-2) showed lingering mortality. Among the laying flocks there was 20-30% drop in hen day egg production which showed slow recovery after 5-6 weeks but failed to reach original production level.

### Pathological studies

#### Gross pathology

**Figure-3:**
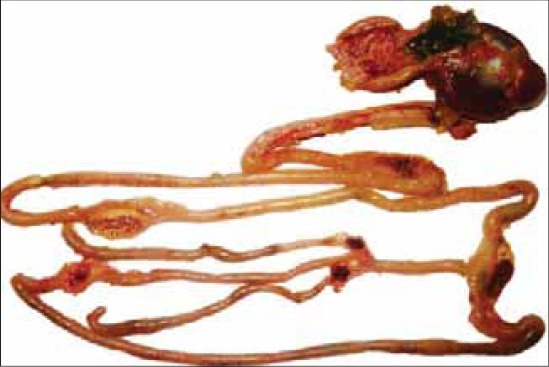
Proventriculus showing diffuse haemorrhages along with haemorrhagic and necrotic patches on intestine and caecal tonsils.

Gross pathological lesions observed in all the layer flocks were typically of velogenic viscerotropic ND. The lesions were characterized by emaciation and dehydration of carcass with deep congestion of breast musculature, multifocal to diffuse haemorrhages around proventricular gland, necrotic (diphtheritic) haemorrhagic ulcers throughout the intestine and caecal tonsils (Figure-3). In addition to the disseminated multiple foci of necrosis, pin-point haemorrhages were observed in the spleen parenchyma. Severe congestion of trachea and lungs was a prominent feature in the majority of layer chicks. In addition, the laying flocks showed degenerated, misshapen and haemorrhagic ova with lesion of egg peritonitis.

### Histopathology

#### Proventriculus

**Figure-4:**
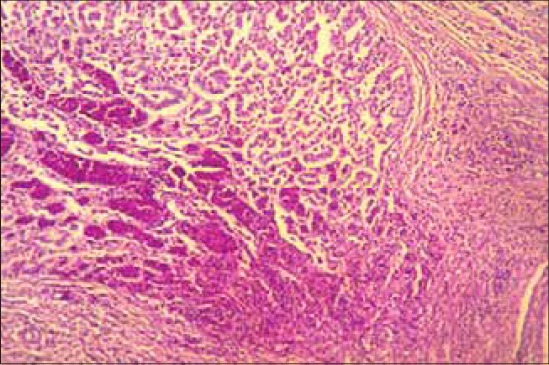
Section of proventriculus from a layer bird showing focal to diffuse haemorrhages in the mucosal and glandular region. H and E stain ×150.

The lesions obtained were focal to diffuse haemorrhages within mucosal ridges as well as in the glandular regions of proventriculus and were suggestive of haemorrhagic proventriculitis (Figure-4). Along with haemorrhages, there was diffuse infiltration of lymphocyte in the mucosa with shortening and sloughing of proventricular papillae.

#### Intestine

Grossly visible diphtheritic and haemorrhagic ulcers revealed lesions like degeneration, necrosis and sloughing of epithelial cells covering from tip of villi to muscular is mucosa along with moderate to severe infiltration of mononuclear cells and focal to diffuse haemorrhages.

#### Caecal tonsils

The lesions in the tonsilar parenchyma revealed multifocal areas of necrosis and hemorrhages and moderate to diffuse infiltration of mononuclear cells, especially lymphocytes in mucosa and submucosa. Some of the sections also showed lymphoid depletion and presence of fibrin debris replacing the lymphoid cells.

#### Spleen

**Figure-5:**
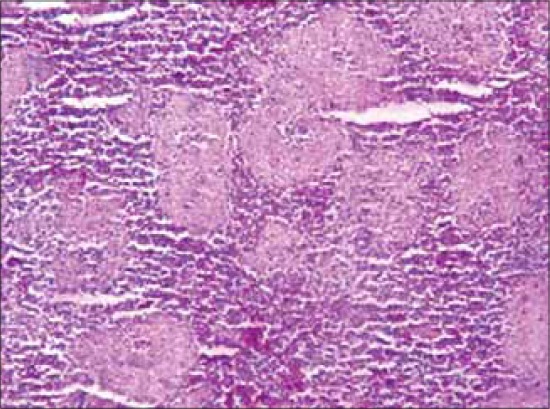
Section of spleen from a layer bird showing mild congestion and multi-focal areas of lymphoid necrosis. H and E stain ×150.

The lesions in the splenic parenchyma includes mild to moderate congestion, multifocal areas of lymphoid necrosis (Figure-5) and reticuloendothelial cell hyperplasia. The lesions were consistent and observed in the majority of flocks.

#### Bursa of fabricius

Bursa of fabricius was collected from layer flocks affected during early growing age. The bursal lesions revealed marked lymphoid necrosis and depletion in follicles with massive heterophilic infiltration in inter and intra-follicular space with cyst formation along with formation of intra-follicular glandular structures with enormous proliferation of fibrous connective tissue in inter-follicular space.

#### Trachea

Tracheal lesions observed during the study were also consistent and seen in majority flocks in Birds. The lesions found in the trachea were variable and included loss of cilia, congestion, oedema and dense infiltration of lymphocytes and macrophages in the mucosa along with desquamation of tracheal mucosal lining epithelium.

#### Lung

The microscopic lesions observed in lung parenchyma were hyperemia and oedema of the parabronchi with infiltration of mononuclear cells. Focal to diffuse areas of haemorrhages were also observed in some of the areas. The lesion observed both in trachea and lungs were indicative of involvement of respiratory tract with much severity during the present outbreaks.

#### Brain

**Figure-6:**
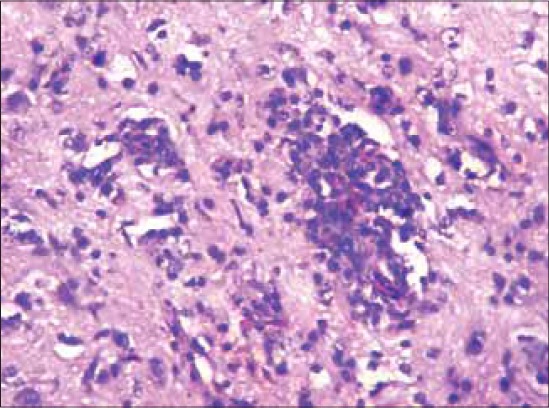
Section of brain from a layer bird showing neuronal degeneration and focal area of gliosis. H and E stain ×300.

Lesions in the brain parenchyma though not consistent but were indicative of non-purulent encephalitis with neuronal degeneration. They included perivascular infiltration of lymphocytes, neuronal degeneration and foci of glial cells in the brain parenchyma (Figure-6). The lesions were observed only in few flocks of grower birds.

### Virus propagation in egg embryo

**Figure-7:**
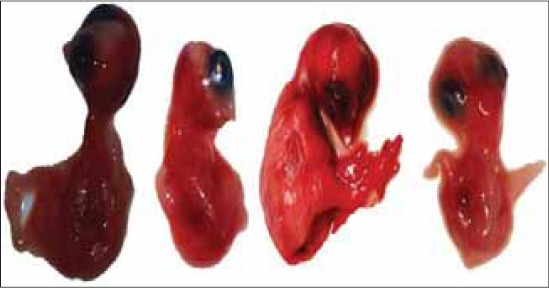
Egg embryos of SPF chicks infected with Newcastle disease virus showing haemorrhagic lesions on the body surface.

All the 13 flock samples (n=13) were inoculated in 9-11 days old embryonated SPF eggs, which resulted in death of all embryos following incubation up to 72 h post-inoculation, thereafter allantois fluid was harvested. The allantois fluid was then subjected to centrifugation at 10000 rpm for 10 min for removal of cellular part. The embryos were taken out one by one in separate petri dishes and washed with nephron sparing surgery for observation of gross lesions produced by the virus. There were multiple haemorrhagic lesions produced throughout the surface of the body of the embryos particularly at the head region (Figure-7).

### HA and HI tests

The test revealed the HA titers in all the 13 field samples and two vaccine sample in between the range of 2^4^-2^8^. HA was confirmed by presence or absence of mat formation of the RBCs (Table-3). The titration was considered as positive at the highest dilution giving complete HA. The positive haemagglutinating allantoic fluids were tested by HI test with known NDV anti-serum and it showed the HI titers of 2^3^-2^9^ (Table-4). The HI titer was considered as the highest dilution of serum causing complete inhibition of 4 HA units of the antigen. The agglutination was assessed by tilting the plates, and streaming of RBCs was compared with control wells (positive-serum, virus and PBS controls) to record the results. Results of HA and HI confirmed the presence of NDV in allantoic fluids obtained from all the 13 of embryonated eggs inoculated with field samples.

**Table-3:** Results of HA test.

Sample ID	No. of wells	HA titre	4 HA unit
NDV 1	6	2^6^ (1:64)	1:16
NDV 2	6	2^6^ (1:64)	1:16
NDV 3	6	2^6^ (1:64)	1:16
NDV 4	6	2^6^ (1:64)	1:16
NDV 5	4	2^4^ (1:16)	1:4
NDV 6	4	2^4^ (1:16)	1:4
NDV 7	6	2^6^ (1:64)	1:16
NDV 8	5	2^5^ (1:32)	1:8
NDV 9	6	2^6^ (1:64)	1:16
NDV 10	6	2^6^ (1:64)	1:16
NDV 11	6	2^6^ (1:64)	1:16
NDV 12	6	2^6^ (1:64)	1:16
NDV 13	8	2^8^ (1:256)	1:64
NDV 14 (F vaccine)	6	2^6^ (1:64)	1:16
NDV 15 (R2B)	6	2^6^ (1:64)	1:16

NDV=Newcastle disease virus, HA=Haemagglutination

**Table-4:** Results of HI test.

Sample ID	No. of wells	HI titre
NDV 1	4	2^4^ (1:16)
NDV 2	3	2^3^ (1:8)
NDV 3	5	2^5^ (1:32)
NDV 4	9	2^9^ (1:512)
NDV 5	6	2^6^ (1:64)
NDV 6	2	2^2^ (1:4)
NDV 7	2	2^2^ (1:4)
NDV 8	5	2^5^ (1:32)
NDV 9	3	2^3^ (1:8)
NDV 10	4	2^4^ (1:16)
NDV 11	3	2^3^ (1:8)
NDV 12	9	2^9^ (1:512)
NDV 13	3	2^3^ (1:8)
NDV 14(F Vaccine)	3	2^3^ (1:8)
NDV 15(R2B)	4	2^4^ (1:16)

NDV=Newcastle disease virus, HI=Haemagglutination inhibition

Along with all the 13, NDV F and R_2_B (Reference strain) allantoic fluid samples were found positive for HA activity. Among these, two samples (15.38%) had HA titer of 1:16 or below, ten samples (76.92%) had titer between 1:16 and 1:64 and one samples (7.69%) had titer of 1:128 and above. Higher titers indicated more concentration of the virus in the respective samples.

HA positive 13 samples and the reference R2B and F strain allantoic fluid samples were subjected to HI test for the confirmation of NDV by using constant antigen (4HA units) and serially diluted known NDV anti-serum. All the 13 samples and the reference R2B and F strain allantoic fluid sample were found positive for HI activity. Among these eight samples (61.53%) had HI titer of 1:16 and below, three samples (23.07%) had titer between 1:16 and 1:64, and two samples (15.38%) had titer between 1:128 and 1:512. This approach was necessary to confirm the specific nature of HA activity. The finding of present study i.e. HA and HI activity of field samples further confirmed that all the 23 flocks were established cases of virulent NDV (VNDV) infection and the disease occurred on these farms in spite of regular prevention programme.

### Pathotyping by ICPI

As per OIE, the ICPI test is scoring systems that evaluate illness or death in chickens. The values in the ICPI test range from 0 to 2.0. The most virulent viruses approach the ICPI value near 2.0, while lentogenic strains are usually close to 0.0. For velogenic strains, the cut-off value of ICPI score is 0.7. The ICPI value found during the present study from all thirteen samples was 2.0 and indicative of velogenic nature of the field NDV strains.

## Discussion

The present findings of the epidemiological studies of the ND outbreaks in layer farms when viewed with the context of earlier workers, gave impression that mortality due to ND in spite of vaccination which included Genotype II virus strain could reach above 50% among layer flocks. The younger flocks appeared more susceptible due to low vaccinal immunity and because of less number of vaccinations. It was also seen that flocks with ND killed vaccination had relatively low mortality. The susceptible age of the disease was found 6-14 weeks among layer flocks, duration of mortality observed was 23 days among layer farms which all above are in agreement with earlier studies carried out by Saidu and Abdu [12] and Gowthaman *et al*. [13]. The disease resulted in significant reduction in body weight and feed consumption both in layer farms and in addition to this drop in egg production (20-30%) in laying flocks is according to beach [14]. The outbreaks were appeared during extreme hot months of April to June in layer flocks. Similar findings were reported by Premavathi and Vardhani [15], Njagi *et al*. [16] and Leow *et al*. [17].

The findings of present field isolates obtained from layer flocks as Genotype XIII (XIIIb) by F gene sequence and whole genome sequence confirmed that mortality and production losses in the present outbreaks were due to highly virulent nature of Genotype XIII pathotype of NDV which supports findings of Tirumurugaan *et al*. [18] who reported that current vaccination programme against ND in layer and broilers which include genotype-II virus strain like Lasota, B_1_ or Komarov does not prevent the clinical disease against velogenic pathotype VII, the present Genotype XIII might have resulted in vaccine failure and resultant outbreaks with heavy mortality.

Similar clinical signs like reduced feed intake, weight loss, depression, somnolence, leg paralysis, watery greenish diarrhoea, and difficult respiration were also reported by Premavathi and Vardhani [15] and Akamura *et al*. [19] during field outbreaks of ND in vaccinated flocks.

Clinical signs such as listlessness, ruffled feathers, prostration, periocularedema, green diarrhea were similar to as reported by Merino *et* *al*. [20] during their experimental study on broilers infected with Quail 2006 (Genotype V) NDV strains following to vaccination with Genotype II NDV strains.

Lesions like multifocal to diffuse haemorrhages around proventricular glands, necrotic haemorrhagic ulcers throughout the intestine and caecal tonsils were similar to the findings observed by Saidu and abdu [12] in 6 weeks old pullets, Gowthaman *et* *al*. [14] who reported disseminated multiple foci of necrosis and pin-point haemorrhages in spleen parenchyma in grower and layer birds affected with virulent Newcastle disease (VND). Pazhanivel *et* *al*. [21] also reported same lesions like severe congestion of trachea and lungs in vaccinated broiler coloured chicken. The gross lesions observed during the present study were also in agreement with the findings of earlier workers Akamura *et* *al*. [19], Wang *et* *al*. [22] and Hu *et* *al*. [23] who reported lesions with more or less severity among vaccinated layer farms.

The histopathological lesions observed in different visceral and lymphoid organs during the present study indicated that the Genotype XIII NDV cause significant pathological lesions in proventriculus, intestine, trachea, lungs as well as lymphoid organs like caecal tonsils, spleen, and bursa of fabricius. Overall nature of histopathological lesions also suggested that present outbreaks caused by Genotype XIII NDV were similar to that of classical viscerotropic velogenic ND. Outbreaks leading to immunosuppression which were in agreement with the findings of Ezema *et* *al*. [24] who reported that VNDV cause marked atrophy of the lymphoid organs, leading to immunosuppression in vaccinated chickens and stated that LaSota vaccination may not protect against the VND in chickens and Wang *et* *al*. [22] who showed severe lymphoid necrosis in lymphoid organ during their study on SPF chickens experimentally infected with JS-5-05-Go (Genotype VIId), ZJ1 (Genotype VIId), XJ-2/97 (Genotype VIId), JS-3-05-Ch (Genotype VIId) strains.

The clinical signs, mortality pattern and postmortem lesions observed during the present outbreaks of ND in layer farms were suggestive of very virulent Newcastle disease. The present available vaccines prepared from Genotype-II NDV probably have failed in protecting flocks from Genotype-XIII NDV infection. Similar method of virus isolation from tissues by using SPF embryonated eggs through intra allantoic inoculation and death of the embryos within 24-96 h of post-inoculation was reported by Tirumurugaan *et* *al*. [25], Diel *et* *al*. [26], Fringe *et* *al*. [27] and Majed *et* *al*. [28] during their study.

In present study 13 clinically suspected layer farms are positive for NDV similar to Majed *et al*. [28], who isolated NDV using SPF chicken embryos through chorio-allantoic membrane route from 26 samples out of 34 clinically diagnosed ND field samples. In the present study the observation of death of all the embryos following inoculation with presence of petechial or ecchymotic haemorrhages throughout the body surface were in accordance with the results of Fringe *et* *al*. [27] and Haque *et* *al*. [29]. The present HA activity results of NDV field isolates were in accordance with the reports of Rakibul Hasan *et* *al*. [30] and Gowthaman *et* *al*. [31].

The present findings of NDV confirmation by HI were in accordance with earlier reports of RakibulHasan *et* *al*. [30] and Tirumurugaan *et* *al*. [18]. In present study 13 HA positive NDV allantoic fluid was also found positive by HI. It is similar to Majed *et* *al*. [28] who reported that out of 26 HA positive NDV suspected allantoic fluid, 19 (73.08%) were positive by HI. The finding of present study i.e. HA and HI activity of field samples further confirmed that all the 13 flocks were established cases of VNDV infection and the disease occurred on these farms in spite of regular prevention programme.

Present study all 13 layer farms found ICPI 2.0 which were characterstics of Velogenic NDV. The results of the present study were in accordance with the previous work of Ananth *et* *al*. [32], Tirumurugaan *et* *al*. [18], Munir *et* *al*. [33], Al-Habeeb *et al*. [34] and Naveen *et al*. [35]. Result was also in accordance to Kianizadeh *et al*. [36] who recovered 12 isolates of NDV from different outbreaks in Iran. They found ICPI varying from 1.7 to 1.96 and described them as characteristic velogenic NDV. Isolation of novel Genotype-XIII from the field outbreaks and ICPI score of 2.0 confirmed that present outbreaks were due to Genotype-XIII NDV which was velogenic in nature.

## Conclusion

Commercial layer farms which showed ND outbreaks had undergone routine preventive ND vaccination programme. The management of these farms was well acquainted with appropriate vaccination procedure. Isolation of Genotype-XIII and its virulent nature from the field outbreaks also confirmed the limitations of the presently available live and inactivated vaccines to protect the flocks against Genotype-XIII NDV infection. The present outbreaks of NDV infection in layer farms were due to velogenic nature of virus Genotype-XIII and presently available vaccines have limited protection against this genotype.

## Authors’ Contribution

This study is the major component of the work towards the M.V.Sc thesis of the first author JHK under the guidance of the second author BPJ and KSP, SP, PDG helped in the experimental investigation and organized the manuscript. DJG and RAM thoroughly revised the same and provided guidance for research work. All authors read and approved the final version of the manuscript.
